# Nitrogen-rich organic soils under warm well-drained conditions are global nitrous oxide emission hotspots

**DOI:** 10.1038/s41467-018-03540-1

**Published:** 2018-03-19

**Authors:** Jaan Pärn, Jos T. A. Verhoeven, Klaus Butterbach-Bahl, Nancy B. Dise, Sami Ullah, Anto Aasa, Sergey Egorov, Mikk Espenberg, Järvi Järveoja, Jyrki Jauhiainen, Kuno Kasak, Leif Klemedtsson, Ain Kull, Fatima Laggoun-Défarge, Elena D. Lapshina, Annalea Lohila, Krista Lõhmus, Martin Maddison, William J. Mitsch, Christoph Müller, Ülo Niinemets, Bruce Osborne, Taavi Pae, Jüri-Ott Salm, Fotis Sgouridis, Kristina Sohar, Kaido Soosaar, Kathryn Storey, Alar Teemusk, Moses M. Tenywa, Julien Tournebize, Jaak Truu, Gert Veber, Jorge A. Villa, Seint Sann Zaw, Ülo Mander

**Affiliations:** 1 Department of Geography, Instute of Ecology and Earth Sciences, University of Tartu, Tartu, 51014 Estonia; 2 School of Geography, Geology and the Environment, Keele University, Newcastle, ST5 5BG UK; 3 School of Geography, Earth and Environmental Sciences, University of Birmingham, Birmingham, B15 2TT UK; 4 Ecology and Biodiversity, Department of Biology, Utrecht University, Utrecht, 3584 CH The Netherlands; 5 Institute of Meteorology and Climate Research, Karlsruhe Institute of Technology, Garmisch-Partenkirchen, 82467 Germany; 6 Centre for Ecology and Hydrology, Edinburgh, EH26 0QB UK; 7 Department of Forest Ecology and Management, Swedish University of Agricultural Sciences, Umeå, SE901 83 Sweden; 8 Natural Resources Institute Finland (Luke), Helsinki, FIN-00790 Finland; 9 Department of Earth Sciences, University of Gothenburg, Gothenburg, SE405 30 Sweden; 10 Institute of Earth Sciences, National Center for Scientific Research (CNRS) and University of Orléans, Orléans, 45100 France; 11 UNESCO Chair of Environmental Dynamics and Climate Change, Yugra State University, Khanty-Mansiysk, 628012 Russia; 12 Atmospheric Composition Research, Finnish Meteorological Institute, Helsinki, FIN-00101 Finland; 13 Department of Botany, Institute of Ecology and Earth Sciences, University of Tartu, Tartu, 51014 Estonia; 14 Everglades Wetland Research Park, Kapnick Center, Florida Gulf Coast University, Naples, 4940 FL USA; 15 Institute of Plant Ecology, Justus Liebig University Giessen, Giessen, 35392 Germany; 16 University College Dublin (UCD) School of Biology and Environmental Science, UCD Earth Institute, Dublin, 4 Ireland; 17 Department of Plant Physiology, Institute of Agricultural and Environmental Sciences, Estonian University of Life Sciences, Tartu, 51014 Estonia; 18 Estonian Fund for Nature, Tartu, 51014 Estonia; 19 School of Geographical Sciences, University of Bristol, Bristol, BS8 1SS UK; 20 Department of Primary Industries, Parks, Water and Environment, Tasmanian Government, Hobart, 7001 TAS Australia; 21 Department of Agricultural Production, College of Agricultural and Environmental Sciences, Makerere University, Kampala, 7062 Uganda; 22 Hydrosystems and Bioprocesses Research Unit, National Research Institute of Science and Technology for Environment and Agriculture (IRSTEA), Antony, 92160 France; 23 Grupo de Investigación Aplicada al Medio Ambiente, Corporacion Universitaria Lasallista, Caldas, 51 118 Colombia; 24 Forest Resource Environment Development and Conservation Association, Yangon, 0951 Myanmar

## Abstract

Nitrous oxide (N_2_O) is a powerful greenhouse gas and the main driver of stratospheric ozone depletion. Since soils are the largest source of N_2_O, predicting soil response to changes in climate or land use is central to understanding and managing N_2_O. Here we find that N_2_O flux can be predicted by models incorporating soil nitrate concentration (NO_3_^−^), water content and temperature using a global field survey of N_2_O emissions and potential driving factors across a wide range of organic soils. N_2_O emissions increase with NO_3_^−^ and follow a bell-shaped distribution with water content. Combining the two functions explains 72% of N_2_O emission from all organic soils. Above 5 mg NO_3_^−^-N kg^−1^, either draining wet soils or irrigating well-drained soils increases N_2_O emission by orders of magnitude. As soil temperature together with NO_3_^−^ explains 69% of N_2_O emission, tropical wetlands should be a priority for N_2_O management.

## Abstract (web summary)

In a global field survey across a wide range of organic soils, the authors find that N_2_O flux can be predicted by models incorporating soil nitrate concentration (NO_3_^–^), water content and temperature. N_2_O emission increases with NO_3_^–^ and temperature and follows a bell-shaped distribution with water content.

## Introduction

Organic soils make up more than one-tenth of the world’s soil nitrogen (N) pool^1^ and are a significant global source of the greenhouse gas nitrous oxide (N_2_O). We do not fully understand the underlying microbial production and consumption processes and how these interact with environmental drivers such as the microclimate, physics, and chemistry of the soil^2^. N_2_O can be emitted as a by-produce of both incomplete nitrification and incomplete denitrification. Under anaerobic conditions, N is primarily conserved in organic compounds, and nitrification (the conversion of ammonium (NH_4_^+^) to NO_3_^−^) is limited to the rooting zone or is absent. The normally low availability of NO_3_^−^ also restricts rates of denitrification (the conversion of NO_3_^−^ to N_2_) in anaerobic soil; if sufficient NO_3_^−^ is present but oxygen remains restricted, denitrification may go to completion, producing atmospheric N_2_^3–6^. Reduction of soil moisture promotes mineralisation of organic N to NH_4_^+^, which can be nitrified to NO_3_^−^^7,8^, and produces the partially-oxidised conditions that are conducive to incomplete denitrification, a major source of N_2_O^9^. N_2_O emission has been both positively and negatively correlated with soil moisture, as water-filled pore space (WFPS) or volumetric water content (VWC)^10–26^ depending upon water status: intermediate levels of around 50–80% WFPS or VWC appear to be optimal for N_2_O production^26–36^.

Increases in soil temperature normally enhance N_2_O production^37^ up to about 24 °C, where bacterial denitrification reaches an optimum^38,39^, above which N_2_O efflux drops. However, denitrifier communities may adapt to higher temperatures, leading to further increases in N_2_O emissions^40^. A review of laboratory and field studies shows inconsistent relationships between temperature and N_2_O emissions^13,21,41^ from strongly positive to negative, illustrating that temperature alone cannot explain N_2_O fluxes but must be considered in the context of other drivers, especially soil moisture. At near-zero soil temperatures, the freeze-thaw effect may produce significant amounts of N_2_O^42–45^.

As growing population pressure has increased the extent of fertilised and drained organic soil, nitrogen-rich organic soils will become increasingly important global N_2_O sources^2,46^. Currently N_2_O contributes 12% of CO_2_-equivalent GHG emissions from land use in tropics^47^. Quantifying the influence of both increasing rates of land drainage and climate change on organic soil N_2_O fluxes is thus critically important^2^. However, emission factors used to assess N_2_O fluxes from different land uses and ecosystems are usually simple proportions of the application rate of fertiliser (or atmospheric reactive N deposition for non-cultivated soils) and broad land-use categories; these models also do not take into account climate-related changes^48^. Thus we lack an inclusive model to quantify the potential of organic soils to be a globally important source of N_2_O^2,49^. To address this challenge we undertook a standardised global survey of in situ N_2_O fluxes from organic soils, together with ancillary measurements of key drivers, to derive a model of N_2_O emissions that would be applicable to a wide range of biomes and environmental conditions. We find that N_2_O emission from organic soils increases with rising soil NO_3_^−^, follows a bell-shaped distribution with soil moisture, and increases with rising soil temperature. This emphasises the importance of warm drained fertile soils  to climate change.

## Results

### Principal component analysis

**Fig. 1:**
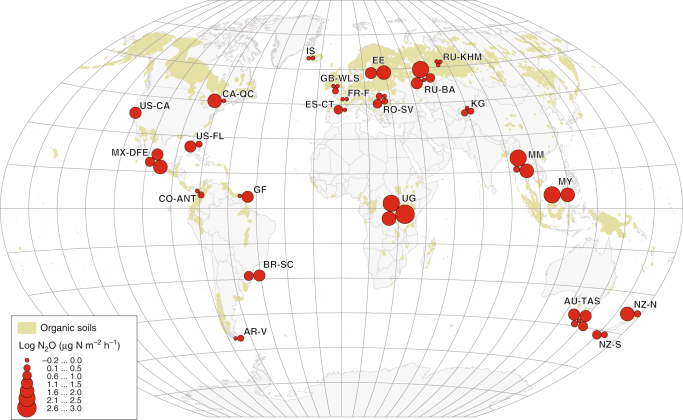
Site-mean N_2_O fluxes by study region superimposed on a global organic-soil map. Country and region codes are defined after ISO 3166-2. The distribution of organic soil was defined as >150 t C_org_ ha^−1^ from the Global Soil Organic Carbon Estimates (courtesy of the European Soil Data Centre) + 0.5 geographical-degrees buffer for visual generalisation

**Fig. 2:**
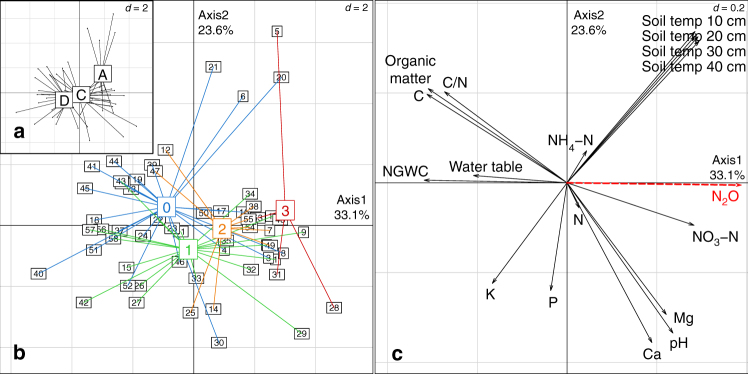
Ordination plots based on principal component analysis grouping sites and variables. **a** Köppen climates (A) tropical, (C) temperate and (D) boreal; **b** intensity of agricultural use (0) no agriculture, (1) moderate grazing or mowing, (2) intensive grazing or mowing and (3) arable; **c** soil physical and chemical parameters. N_2_O emission used as passive variable. *d*: grid scale, VWC volumetric water content. See Supplementary Data 1 for site names

Site-mean N_2_O fluxes by study region superimposed on a global organic-soil map are shown in Fig. 1. The principal component analysis (PCA) differentiated tropical sites from temperate and boreal ones, and low agricultural-intensity sites (index 0 and 1) from arable sites (index 3) (*p* < 0.05; Fig. 2a, b). Soil NO_3_^−^ was positively related to N_2_O emission; VWC and water table were strongly negatively correlated with N_2_O emissions, and C/N, C, and organic matter were less strongly negatively correlated with N_2_O emissions, and soil temperature was positively related to N_2_O emissions (Fig. 2c). Soil-available P was orthogonal to the N_2_O-flux vectors, indicating no correlation. The difference between N_2_O emissions from drained and natural sites was clear in all three major climate types (Supplementary Table 1).

### Global models

**Fig. 3:**
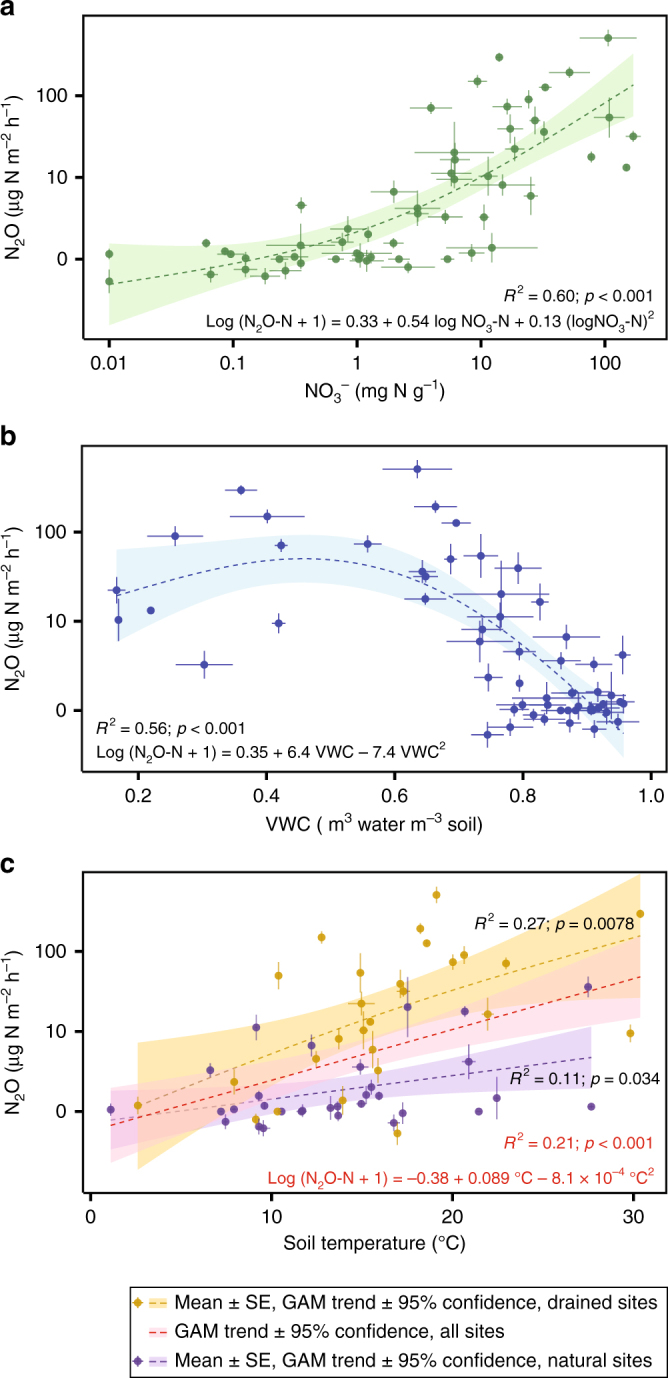
Relationships between site-mean N_2_O fluxes and soil parameters. The panels correspond to the relationships between N_2_O fluxes and: **a** nitrate; **b** volumetric water content; **c** soil temperature at 40 cm depth across all sites, and drained and natural sites. The error bars correspond to standard errors of the mean (s.e.m.). *N* = 58

**Fig. 4:**
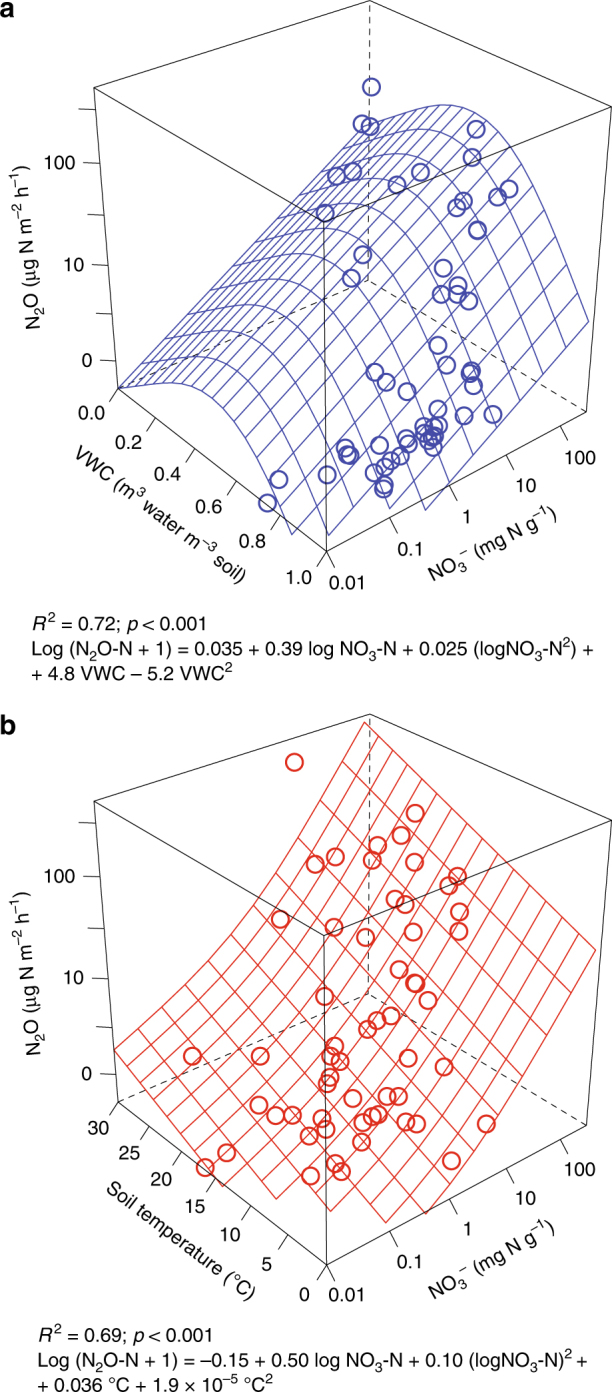
Site-mean N_2_O flux multiple-regression models. **a** Soil nitrate and volumetric water content (VWC); **b** soil nitrate and temperature at 40 cm depth. *N* = 58

Of the 18 parameters assessed (Supplementary Data 1), soil NO_3_^−^ was the strongest predictor of site-mean N_2_O, explaining 60% of the variation in log N_2_O flux (Fig. 3a). The generalised additive model (GAM) trend was similar to concave log-log quadratic. Inclusion of site-mean VWC (Fig. 3b) raised the explanatory power of the multiple-regression GAM to 72% (*n* = 58; *R*^2^ = 0.72; *p* < 0.001; Eq. (1); Fig. 4a). The regression surface was similar to a convex paraboloid with an apex at approximately 50% VWC:

1 $$\begin{array}{l}{\rm Log}\left( {{\rm N}_{2}{{\rm O \text{-} N}} + 1} \right) 	= 0.035 + 0.39\,{{\rm logNO}_{3}}\text{-}{\rm N} \\ 	+ 0.025\left( {{{\rm logNO}_{3}}\text{-}{\rm N}} \right)^2\\ 	+ 4.8\,{{\rm VWC}}-5.2\,{{\rm VWC}}^2\end{array}$$

**Fig. 5:**
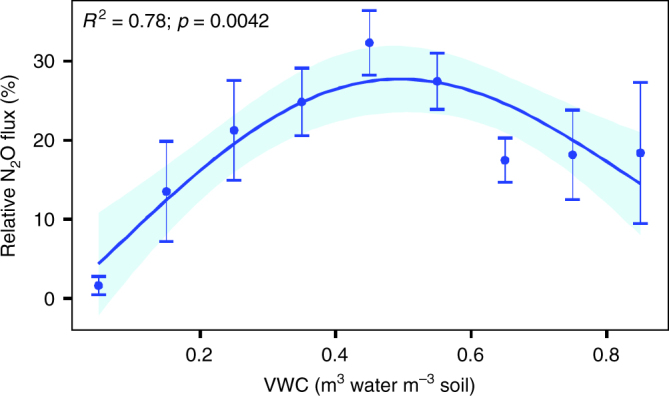
Relative N_2_O fluxes versus volumetric water content (VWC) in 11 published annual time series. The N_2_O fluxes are scaled to the maximum value measured at each respective site. The dots and whiskers are average ± s.e.m. within the respective soil-moisture class. The curve is the GAM regression (*k* = 3) between average relative N_2_O fluxes and VWC. The light blue area marks the 95% confidence limits of the regression line

In an independent comparison of the model with published data, relative N_2_O emissions were represented well. The relationship between the mean N_2_O fluxes (relative to the maximum value in the respective external data set) and VWC was best described by a bell-shaped GAM regression curve (*R*^2^ = 0.78; *p* < 0.001; Fig. 5) similar to the VWC component of our global model (Fig. 3b). Both curves peaked at around 50% soil moisture.

Both our model and the literature support the idea that fluctuation around the intermediate VWC (~0.5 m^3^ m^−3^) creates variability in the oxygen content within the soil profile. That, in turn, stimulates mineralisation and nitrification which contribute to higher NO_3_^−^ content^8,9,50,51^. Intermediate VWC also promotes incomplete denitrification, in agreement with early conceptualisations^25,40^, previous regional-scale studies^28,33–35^ and experiments^9,51,52^. The maximum N_2_O emission at the intermediate VWC means that both wetting from lower moisture values and drying from higher moisture will increase N_2_O emissions. At a VWC of ~0.8 m^3^ m^−3^, oxygen concentration in the pore water is 5–9% of saturation, which is low enough to trigger N_2_O production but insufficient for complete denitrification^9,51,52^.

There was no significant relationship between N_2_O flux and NH_4_-N among our observations (*p* = 0.79), suggesting that denitrification was probably the main source of N_2_O emissions rather than nitrification. Only one site (Tasmania drained fen 2) directly received mineral fertiliser, whereas the nitrate in the other 57 sites originated from livestock and natural sources such as nitrification, atmospheric deposition, runoff and groundwater. Thus our global model describes N_2_O emission due to grazing and naturally transported nitrate.

We found only a weak relationship between N_2_O fluxes and soil temperature (40cm-depth temperature log GAM *R*^2^ = 0.21, *p* < 0.001; Fig. 3c). The soil temperatures normalised to local annual air-temperature maxima gave even lower correlation values (e.g. with temperature at 40 cm-depth log GAM *R*^2^ = 0.09, *p* = 0.018). This may have been partially due to the short time span of our measurements per site. However, that is consistent with the meta-analysis of published data in eleven papers showing no correlation between long-term N_2_O fluxes and soil temperature^17,24,31,32,34,53–58^. The test for an upper boundary^59^ in our temperature data was negative (*p* > 0.05). Therefore we accepted the H_0_ hypothesis that our data are from a bivariate normal process and so the envelope of the data points does not represent a boundary. This also suggests that the high N_2_O fluxes were measured in soils where temperature was not the limiting factor. A multiple-regression GAM model containing soil temperature at 40 cm depth and log NO_3_^−^ explained 69% of log N_2_O fluxes (*n* = 58; *R*^2^ = 0.69; *p* < 0.001; Eq. (2); Fig. 4b):

2 $$\begin{array}{l}{{\rm Log}}\left( {{{\rm N}}_2{{\rm O\text{-}\mathrm{N}}} + 1} \right) 	= -0.15-0.50\,{{\rm logNO}}_3\text{-}\mathrm{N} \\ 	+ 0.10\left( {{{\rm logNO}}_3\text{-}\mathrm{N}} \right)^2 \\ 	+ 0.036\,{}^ \circ {{\rm C}} + 1.9\times10^{-5}{} ^ \circ{{\rm C}}^2\end{array}$$

Within our drained sites (Supplementary Data 1; *n* = 27) the temperature relationship was somewhat stronger (*R*^2^ = 0.27; *p* < 0.0078; Fig. 3c). This shows that organic soils exposed to warmer conditions, such as in the tropics, can act as N_2_O-emission hotspots where soil moisture is optimal (Fig. 3b) and NO_3_^−^ is above a threshold of 5 mg N kg^−1^ (Fig. 3a).

Because we sampled each site for only a few days and that we visited temperate and boreal sites during the growing seasons this study was not designed to detect the effect of seasonal or synoptic-scale variation of temperature, soil nitrate, and other factors within each site. Thus our global models are only applicable to estimate daily N_2_O emissions based on instantaneous environmental conditions at organic-soil sites. Annual-average N_2_O emissions at sites under a seasonal climate may be more difficult to draw from our model. Yet the model could be useful to estimate N_2_O emissions at sites under a lack of seasonal variation in environmental conditions such as the humid tropical climate. Upscaling our three tropical sites with intensive land use (the Malaysian oil palm plantation, and the Myanmar and Uganda arable sites; Supplementary Data 1) to a year’s duration and comparing them with the special default emission values (EF2) in IPCC Guidelines 2006 for tropical organic soils^60^ (16 kg N_2_O-N ha^−1^ y^−1^, range 5–48 kg ha^−1^ y^−1^) gave us the following results. For the Malaysian site, soil temperature was 27–30 °C, the mean emission rate was 294.3 μg N_2_O-N m^−2^ h^−1^ = 25.8 kg N_2_O-N ha^−1^ y^−1^. For Myanmar, 14–19 °C (upland), the figures were 125.5 μg N_2_O-N m^−2^ h^−1^ = 11.0 kg N_2_O-N ha^−1^ y^−1^. For Uganda, 17–20 °C (upland), the figures were 507.3 μg N_2_O-N m^−2^ h^−1^ = 44.5 kg ha^−1^ y^−1^. Thus the annual fluxes obtained by this simple upscaling all fell within the IPCC tropical default range.

### Other potential drivers

The logarithm of C:N ratio, a common scalar explanatory variable used to predict N_2_O emissions^61^, was correlated with N_2_O emissions (*R*^2^ = 0.16; *p* = 0.001; Supplementary Fig. 1) but was not significant in a model that contained NO_3_^−^. Agricultural intensity explained 25% of the variability in N_2_O fluxes (log GAM *R*^2^ = 0.25; *p* < 0.001), but again was not significant in a model containing NO_3_^−^ and VWC as proximal controllers of N_2_O emission. The effect of agriculture on N_2_O emissions was mainly related to cultivation (Fig. 2b). We could detect no significant difference between N_2_O emissions from agriculturally unused sites and pastures or hay fields. Thus non-agricultural sources of elevated N (e.g. from chronically elevated atmospheric N deposition), and lower soil water content (e.g. reductions in precipitation) would likely have a similar impact on N_2_O emissions as agricultural fertilisation and drainage.

## Discussion

This is the first time that simple, robust global models of N_2_O emissions driven by nitrate, moisture and temperature of organic soils have been identified. It is notable that the models encompass temperate, continental, and tropical biomes. Our findings provide more accurate models of the drivers of N_2_O emissions from organic soils across a wide range of biomes and management regimes than heretofore developed. This highlights the importance of soil nitrate, moisture, and temperature in organic soils as significant global contributors to climate change and stratospheric ozone depletion. Our global-scale models show that constantly high soil moisture results in low N_2_O emissions, whereas drainage creates fluctuation around the intermediate soil moisture and thus increases N_2_O emissions from organic soils. The temperature effect on N_2_O emissions emphasises the importance of considering the warm fertile soils in the global N_2_O budget. The implication of this work is that wetland conservation should be a priority for climate change mitigation, particularly given the evidence for future increases in the magnitude and frequency of summer droughts^60^. The anticipated large N_2_O emissions from N-rich drained organic soils can be mitigated through wetland conservation and restoration, and through appropriate soil management, such as reduced tillage, nutrient management and improved crop rotations^46^. These have been implemented to some extent in developed countries but need to be further expanded and extended, as a matter of urgency, to tropical and sub-tropical regions.

## Methods

### Study sites

Our global soil- and gas-sampling campaign was conducted during the vegetation periods between August 2011 and March 2017, following a standard protocol. We sampled 58 organic-soil sites using criteria for organic soils (>12% soil carbon content in the upper 0.1 m) adapted from the FAO World Reference Base for Soils^62^ in 23 regions throughout the A (rainy tropical), C (temperate), and D (boreal) climates of the Köppen classification (Fig. 1; Supplementary Data 1). Both natural and artificially drained sites were identified, based on the proximity of drainage ditches, water table height, and characteristic vegetation. The hydrology and trophic status of the natural sites ranged from groundwater-fed swamps and fens to ombrotrophic peat bogs. We measured the most important environmental drivers that were possible.

### Field and laboratory measurements

Within each region, we established sites to capture the full range of environmental conditions as described above. The depth of the topsoil organic horizon ranged from 0.1 to 6 m across the sites. Land use ranged from natural mire and swamp forest to managed grassland and arable land. A four-grade agricultural-intensity index was used to quantify the effect of land conversion: 0—no agricultural land use (natural mire, swamp, or bog forest), 1—moderate grazing or mowing (once a year or less), 2—intensive grazing or mowing (more than once a year), and 3—arable land (directly fertilised or unfertilised). The agricultural intensity index was estimated based on observation and contacts with site managers and local researchers.

At each site, 1 to 4 stations were established 15–500 m apart to maximise the environmental variance. Each station was instrumented with 3–5 white opaque PVC 65 L truncated conical chambers 1.5–5 m apart and a 1-m-deep observation well (a 50-mm-diametre perforated PP-HT pipe wrapped in geotextile). The total number of chambers was 444. N_2_O fluxes were measured using the static chamber method^63^ using PVC collars of 0.5 m diameter and 0.1 m depth installed in the soil. A stabilisation period of 3–12 h was allowed before gas sampling to reduce the disturbance effect on fluxes from inserting the collars. The chambers were placed into water-filled rings on top of the collars. Gas was sampled from the chamber headspace into a 50 mL glass vial every 20 min during a 1-h session. The vials had been evacuated in the laboratory 2–6 days before the sampling. At least three sampling sessions per location were conducted over 3 days. The gas samples were brought to the University of Tartu and analysed for N_2_O concentration within 2 weeks using two Shimadzu GC-2014 gas chromatographs equipped with ECD, TCD, and Loftfield-type autosamplers^63^. N_2_O fluxes were determined on the basis of linear regressions obtained from consecutive N_2_O concentrations in three to five samples taken when the chamber was closed, resulting in 61 negative and 502 positive N_2_O fluxes (*p* < 0.05 for the goodness of fit to linear regression). There were 982 additional insignificant fluxes (*p* > 0.05) below the gas-chromatography measuring accuracy (regression change of N_2_O concentration, δv, <10 ppb) reported as zero in the database and included in the analyses.

Water-table height was recorded daily from the observation wells during the gas sampling at least 8 h after placement. Soil temperature was measured at 10, 20, 30, and 40 cm depth. Soil samples of 150–200 g were collected from the chambers at 0–10 cm depth after the final gas sampling. Humification was rated on the von Post scale, 1 to 10 grades from completely undecomposed to completely decomposed peat^64^. The soil samples were brought to Estonian University of Life Sciences for chemical and physical analyses. During transport, the samples were kept below the ambient soil temperature at which they were collected.

In the laboratory, plant-available phosphorus (P) was determined on a FiaStar5000 flow-injection analyser (KCl extractable). Plant-available potassium (K) was determined from the same solution by the flame-photometric method, and plant-available magnesium (Mg) was determined from a 100-mL NH_4_-acetate solution with a titanium-yellow reagent on the flow-injection analyser. Available calcium (Ca) was analysed using the same solution by the flame-photometrical method. Soil pH was determined on a 1 N KCl solution^65^. Soil NH_4_-N and NO_3_-N were determined on a 2 M KCl extract of soil by flow-injection analysis^65^. Total nitrogen and carbon contents of oven-dry samples were determined using a dry-combustion method on a varioMAX CNS elemental analyser. The organic-matter content of oven-dry soil (SOM) was determined by loss on ignition at 360 °C. We determined gravimetric water content (GWC) as the difference between the fresh and oven-dry weight divided by the oven-dry weight^66^. Bulk density was determined as follows^67^:

3 $${{\rm BD}} = \left( {D_{{{\rm bm}}}\cdot {{D}}_{{{\rm bo}}}} \right)/\left( {{{\rm SOM}}\cdot {{D}}_{{{\rm bm}}} + \left( {1-{{\rm SOM}}} \right)\cdot {{D}}_{{{\rm bo}}}} \right),$$

where:

BD is bulk density, g cm^−3^,

*D*_bm_ is the empirically determined bulk density of the mineral fraction (2.65 g cm^−3^)^66^,

*D*_bo_ is the empirically determined bulk density of the organic fraction (0.035–0.23 g cm^−3^ according to the von Post humification scale^68^), and

SOM is the organic content of the oven-dry soil, g g^−1^.

We determined VWC as^66^:

4 $${{\rm VWC}} = {{\rm GWC}}\cdot {{\rm BD}},$$

where:

VWC is volumetric water content, m^3^ m^−3^,

GWC is gravimetric water content, Mg Mg^−1^, and

BD is bulk density, Mg m^−3^.

For normalising the soil temperature to possible local optima we divided our soil-temperature measurements with the mean air temperature at the nearest weather station in the warmest month of the year^69^ (KNMI Climate Explorer http://climexp.knmi.nl; Supplementary Data 1).

### Statistical analysis

Principal component analysis (PCA), Spearman’s rank correlation and stepwise multiple regression of site-mean efflux vs. the environmental parameters were used. The tests were run using both untransformed and log-transformed N_2_O fluxes. Before the log-transformation, a constant value was added to all fluxes to account for negative values. Normality of the variables and the residuals was checked by the Shapiro–Wilk test. Neither the N_2_O fluxes nor their logarithms were normally distributed (*p* < 0.05); this is a commonly reported issue with N_2_O. Therefore only a nonparametric test such as Spearman’s rank correlation and generalised additive models (GAM) could be applied. We used the mgcv package of the R Project to calculate the GAM regressions using minimal smoothness (*k* = 3). We reported *p*-values (significance level *p* < 0.05) from the summaries of the GAM regressions produced by the summary.gam package of the R Project. We only reported GAM regressions when the residuals were normally distributed. As a presumption for the stepwise multiple regression, the independent variables were checked for GAM concurvity—we only reported multiple relationships with a variance inflation factor <10 between the independent variables. We tested the presence of a boundary in our data^59^. The test compared the density of points in the region of the data set’s upper envelope to the expected density of the upper envelope of a bivariate normally distributed data set of the same size^59^.

### Literature analysis

In order to compare our model with independent external data, we surveyed literature referenced in the Thomson Reuters Web of Science. The search terms were: N_2_O and organic soil and nitrous oxide and organic soil. We only included publications that reported time series of at least a year’s duration that reported N_2_O fluxes and simultaneous soil temperature and soil moisture observations (either VWC or WFPS). Eleven papers^17,24,31,32,34,53–58^ qualified under these criteria. The study sites were fairly evenly distributed throughout major organic soil regions of the world. Only three of these papers reported soil NO_3_^−^ concentrations^17,24,58^. We converted the WFPS values to VWC as follows^66^:

5 $${{\rm VWC}} = {{\rm WFPS}}\cdot {{\rm TP}},$$

where:

VWC is volumetric water content, m^3^ water m^−3^ fresh soil,

WFPS is water-filled porosity, m^3^ water m^−3^ pore space, and

TP is total porosity, m^3^ pore space m^−3^ soil.

To standardise the highly different absolute N_2_O values among data sets we normalised them by scaling to the maximum value measured at each site^70^. We calculated average relative N_2_O fluxes in 15 soil temperature classes: 0 °C to 2 °C, 2 °C to 4 °C, … and 28 °C to 30 °C, and 10 soil moisture classes: 0% to 10%, 10% to 20%,… and 90% to 100%. Linear and GAM regressions with minimal smoothness (*k* = 3) were determined between soil temperature, soil moisture and both the individual and average relative N_2_O fluxes.

### Data availability

The data reported in this paper are deposited in the PANGAEA repository https://doi.pangaea.de/10.1594/PANGAEA.885897.

## Electronic supplementary material

Supplementary Information

Peer Review File

Description of Additional Supplementary Files

Supplementary Data 1

## References

[1] Batjes NH (2014). Total carbon and nitrogen in the soils of the world. Eur. J. Soil Sci..

[2] Butterbach-Bahl K, Baggs EM, Dannenmann M, Kiese R, Zechmeister-Boltenstern S (2013). Nitrous oxide emissions from soils: how well do we understand the processes and their controls?. Philos. Trans. R. Soc. Lond. B Biol. Sci..

[3] Jones CM, Stres B, Rosenquist M, Hallin S (2008). Phylogenetic analysis of nitrite, nitric oxide, and nitrous oxide respiratory enzymes reveal a complex evolutionary history for denitrification. Mol. Biol. Evol..

[4] Meng W, Moore TR, Talbot J, Pierre JHR (2014). The cascade of C:N:P stoichiometry in an ombrotrophic peatland: from plants to peat. Environ. Res. Lett..

[5] Shcherbak I, Millar N, Robertson GP (2014). Global metaanalysis of the nonlinear response of soil nitrous oxide (N_2_O) emissions to fertilizer nitrogen. Proc. Natl Acad. Sci. USA.

[6] Meng W, Moore TR, Talbot J, Riley JL (2015). The stoichiometry of carbon and nutrients in peat formation. Glob. Biogeochem. Cycl..

[7] Martikainen PJ, Nykänen H, Crill P, Silvola J (1993). Effect of a lowered water table on nitrous oxide fluxes from northern peatlands. Nature.

[8] Golovchenko AV, Tikhonova EY, Zvyagintsev DG (2007). Abundance, biomass, structure, and activity of the microbial complexes of minerotrophic and ombrotrophic peatlands. Microbiology.

[9] Rubol S, Silver WL, Bellin A (2012). Hydrologic control on redox and nitrogen dynamics in a peatland soil. Sci. Total Environ..

[10] Clayton H, McTaggart IP, Parker J, Swan L, Smith KA (2014). Nitrous oxide emissions from fertilised grassland: a 2-year study of the effects of N fertiliser form and environmental conditions. Biol. Fertil. Soils.

[11] Skiba UM, Sheppard LJ, Macdonald J, Fowler D (1998). Some key environmental variables controlling nitrous oxide emissions from agricultural and semi-natural soils in Scotland. Atmos. Envir.

[12] Dobbie KE, McTaggart IP, Smith KA (1999). Nitrous oxide emissions from intensive agricultural systems: variations between crops and seasons, key driving variables, and mean emission factors. J. Geophys Res Atmos..

[13] Dobbie KE, Smith KA (2001). The effects of temperature, water-filled pore space and land use on N_2_O emissions from an imperfectly drained gleysol. Eur. J. Soil Sci..

[14] Dobbie KE, Smith KA (2003). Nitrous oxide emission factors for agricultural soils in Great Britain: the impact of soil water-filled pore space and other controlling variables. Glob. Change Biol..

[15] Pihlatie M, Syväsalo E, Simojoki A, Esala M, Regina K (2004). Contribution of nitrification and denitrification to N_2_O production in peat, clay and loamy sand soils under different soil moisture conditions. Nutr. Cycl. Agroecosyst.

[16] Dobbie KE, Smith KA (2006). The effect of water table depth on emissions of N_2_O from a grassland soil. Soil Use Manag..

[17] Takakai F (2006). Effects of agricultural land-use change and forest fire on N_2_O emission from tropical peatlands, Central Kalimantan, Indonesia. Soil Sci. Plant Nutr..

[18] Couwenberg J, Dommain R, Joosten H (2010). Greenhouse gas fluxes from tropical peatlands in South-East Asia. Glob. Change Biol..

[19] Schaufler G (2010). Greenhouse gas emissions from European soils under different land use: effects of soil moisture and temperature. Eur. J. Soil Sci..

[20] Lesschen JP, Velthof GL, de Vries W, Kros J (2011). Differentiation of nitrous oxide emission factors for agricultural soils. Environ. Pollut..

[21] Teh YA (2011). Large greenhouse gas emissions from a temperate peatland pasture. Ecosystems.

[22] Toma Y (2011). Nitrous oxide emission derived from soil organic matter decomposition from tropical agricultural peat soil in central Kalimantan, Indonesia. Soil Sci. Plant Nutr..

[23] van der Weerden TJ, Kelliher FM, de Klein CAM (2012). Influence of pore size distribution and soil water content on nitrous oxide emissions. Soil Res..

[24] Weslien P, Rutting T, Kasimir-Klemedtsson A, Klemedtsson L (2012). Carrot cropping on organic soil is a hotspot for nitrous oxide emissions. Nutr. Cycl. Agroecosys..

[25] Davidson EA, Keller M, Erickson HE, Verchot LV, Veldkamp E (2000). Testing a conceptual model of soil emissions of nitrous and nitric oxides. Bioscience.

[26] Schmidt U, Thöni H, Kaupenjohann M (2000). Using a boundary line approach to analyze N_2_O flux data from agricultural soils. Nutr. Cycl. Agroecosys..

[27] Inubushi K, Furukawa Y, Hadi A, Purnomo E, Tsuruta H (2003). Seasonal changes of CO_2_, CH_4_ and N_2_O fluxes in relation to land-use change in tropical peatlands located in coastal area of South Kalimantan. Chemosphere.

[28] Ball T, Smith KA, Moncrieff JB (2007). Effect of stand age on greenhouse gas fluxes from a Sitka spruce *Picea sitchensis* (Bong.) Carr. chronosequence on a peaty gley soil. Glob. Change Biol..

[29] Goldberg SD, Knorr KH, Blodau C, Lischeid G, Gebauer G (2010). Impact of altering the water table height of an acidic fen on N_2_O and NO fluxes and soil concentrations. Glob. Change Biol..

[30] Toma Y (2010). Effects of environmental factors on temporal variation in annual carbon dioxide and nitrous oxide emissions from an unfertilized bare field on Gray Lowland soil in Mikasa, Hokkaido, Japan. Soil Sci. Plant Nutr..

[31] Christiansen JR, Gundersen P (2011). Stand age and tree species affect N_2_O and CH_4_ exchange from afforested soils. Biogeosciences.

[32] Christiansen JR, Vesterdal L, Gundersen P (2012). Nitrous oxide and methane exchange in two small temperate forest catchments-effects of hydrological gradients and implications for global warming potentials of forest soils. Biogeochemistry.

[33] Balaine N (2013). Changes in relative gas diffusivity explain soil nitrous oxide flux dynamics. Soil Sci. Soc. Am. J..

[34] Benanti G, Saunders M, Tobin B, Osborne B (2014). Contrasting impacts of afforestation on nitrous oxide and methane emissions. Agric. For. Meteorol..

[35] Leppelt T (2014). Nitrous oxide emission budgets and land-use-driven hotspots for organic soils in Europe. Biogeosciences.

[36] Sgouridis F, Ullah S (2015). Relative magnitude and controls of in situ N_2_ and N_2_O fluxes due to denitrification in natural and seminatural terrestrial ecosystems using ^15^N tracers. Envir Sci. Tech..

[37] Holtan-Hartwig L, Dörsch P, Bakken LR (2002). Low temperature control of soil denitrifying communities: kinetics of N_2_O production and reduction. Soil Biol. Biochem.

[38] Rysgaard S, Glud RN, Risgaard-Petersen N, Dalsgaard T (2004). Denitrification and anammox activity in Arctic marine sediments. Limnol. Oceanogr..

[39] Jauhiainen J, Kerojoki O, Silvennoinen H, Limin S, Vasander H (2014). Heterotrophic respiration in drained tropical peat is greatly affected by temperature—a passive ecosystem cooling experiment. Environ. Res. Lett..

[40] Farquharson R, Baldock J (2008). Concepts in modelling N_2_O emissions from land use. Plant Soil.

[41] Dijkstra FA (2012). Effects of elevated carbon dioxide and increased temperature on methane and nitrous oxide fluxes: evidence from field experiments. Front. Ecol. Envir.

[42] Koponen HT, Martikainen PJ (2004). Soil water content and freezing temperature affect freeze-thaw related N_2_O production in organic soil. Nutr. Cycl. Agroecosys..

[43] Matzner E, Borken W (2008). Do freeze-thaw events enhance C and N losses from soils of different ecosystems? A review. Eur. J. Soil Sci..

[44] Pihlatie MK (2010). Greenhouse gas fluxes in a drained peatland forest during spring frost-thaw event. Biogeosciences.

[45] Wagner-Riddle, C. et al. Globally important nitrous oxide emissions from croplands induced by freeze-thaw cycles. *Nat. Geosci. ***10**, 279–283 (2017).

[46] Paustian K (2016). Climate-smart soils. Nature.

[47] Roman-Cuesta RM (2016). Hotspots of gross emissions from the land use sector: patterns, uncertainties, and leading emission sources for the period 2000–2005 in the tropics. Biogeosciences.

[48] Alm J (2007). Emission factors and their uncertainty for the exchange of CO_2_, CH_4_ and N_2_O in Finnish managed peatlands. Boreal Environ. Res..

[49] Syakila A, Kroeze C (2011). The global nitrous oxide budget revisited. Greenh. Gas. Meas. Manag..

[50] Kasimir-Klemedtsson A (1997). Greenhouse gas emissions from farmed organic soils: a review. Soil Use Manag..

[51] Linn DM, Doran JW (1984). Effect of water-filled pore-space on carbon-dioxide and nitrous-oxide production in tilled and nontilled soils. Soil Sci. Soc. Am. J..

[52] Liang LL, Grantz DA, Jenerette GD (2016). Multivariate regulation of soil CO_2_ and N_2_O pulse emissions from agricultural soils. Glob. Change Biol..

[53] Berglund O, Berglund K (2011). Influence of water table level and soil properties on emissions of greenhouse gases from cultivated peat soil. Soil Biol. Biochem..

[54] Christiansen JR, Gundersen P, Frederiksen P, Vesterdal L (2012). Influence of hydromorphic soil conditions on greenhouse gas emissions and soil carbon stocks in a Danish temperate forest. For. Ecol. Manag..

[55] Renou-Wilson F, Barry C, Muller C, Wilson D (2014). The impacts of drainage, nutrient status and management practice on the full carbon balance of grasslands on organic soils in a maritime temperate zone. Biogeosciences.

[56] Beyer C, Liebersbach H, Hoper H (2015). Multiyear greenhouse gas flux measurements on a temperate fen soil used for cropland or grassland. J. Plant Nutr. Soil Sci..

[57] He H (2016). Factors controlling nitrous oxide emission from a spruce forest ecosystem on drained organic soil, derived using the CoupModel. Ecol. Model.

[58] Sgouridis F, Ullah S (2017). Soil greenhouse gas fluxes, environmental controls and the partitioning of N_2_O sources in UK natural and semi-natural land use types. J. Geophys Res..

[59] Milne AE, Wheeler HC, Lark RM (2006). On testing biological data for the presence of a boundary. Ann. Appl. Biol..

[60] IPCC. *2006 IPCC Guidelines for National Greenhouse Gas Inventories*(Institute for Global Environmental Strategies, Hayama, 2006).

[61] Klemedtsson L, Von Arnold K, Weslien P, Gundersen P (2005). Soil CN ratio as a scalar parameter to predict nitrous oxide emissions. Glob. Change Biol..

[62] FAO. World reference base for soil resources (FAO, Rome, 2007).

[63] Mander Uuml (2014). Isotopologue ratios of N_2_O and N_2_ measurementsunderpin the importance of denitrification in differently N-loaded riparian alder forests. Environ. Sci. Tech..

[64] Scherbatzkoy, M. N., Edwards, A. C., & Williams, B. L. in *Visual Soil Evaluation: Realizing Potential Crop Production with Minimum Environmental Impact* (eds Ball, B. C. & Munkholm, L. J.) 98 (CABI, Wallingford, UK 2015).

[65] Ruzicka, J. & Hansen, E. H. *Flow Injection Analysis* (J. Wiley & Sons, Wallingford, UK 1981).

[66] McLaren, R. G. & Cameron, K. C. *Soil Science: Sustainable Production and Environmental Protection* (Oxford University Press, Wallingford, UK 2012).

[67] Périé C, Ouimet R (2008). Organic carbon, organic matter and bulk density relationships in boreal forest soils. Can. J. Soil Sci..

[68] Silc T, Stanek W (1976). Bulk density estimation of several peats in northern Ontario using the von Post humification scale. Can. J. Soil Sci..

[69] Parton WJ (2001). Generalized model for NO_x_ and N_2_O emissions from soils. J. Geophys. Res. Atmos..

[70] Machefert SE, Dise NB (2004). Hydrological controls on denitrification in riparian ecosystems. Hydrol. Earth Sys. Sci. Discuss..

