# Author Correction: Nitrogen-rich organic soils under warm well-drained conditions are global nitrous oxide emission hotspots

**DOI:** 10.1038/s41467-018-04197-6

**Published:** 2018-04-26

**Authors:** Jaan Pärn, Jos T. A. Verhoeven, Klaus Butterbach-Bahl, Nancy B. Dise, Sami Ullah, Anto Aasa, Sergey Egorov, Mikk Espenberg, Järvi Järveoja, Jyrki Jauhiainen, Kuno Kasak, Leif Klemedtsson, Ain Kull, Fatima Laggoun-Défarge, Elena D. Lapshina, Annalea Lohila, Krista Lõhmus, Martin Maddison, William J. Mitsch, Christoph Müller, Ülo Niinemets, Bruce Osborne, Taavi Pae, Jüri-Ott Salm, Fotis Sgouridis, Kristina Sohar, Kaido Soosaar, Kathryn Storey, Alar Teemusk, Moses M. Tenywa, Julien Tournebize, Jaak Truu, Gert Veber, Jorge A. Villa, Seint Sann Zaw, Ülo Mander

**Affiliations:** 1 Department of Geography, Instiute of Ecology and Earth Sciences, University of Tartu, 51014 Tartu, Estonia; 2 School of Geography, Geology and the Environment, Keele University, Newcastle, ST5 5BG UK; 3 School of Geography, Earth and Environmental Sciences, University of Birmingham, Birmingham, B15 2TT UK; 4 Ecology and Biodiversity, Department of Biology, Utrecht University, 3584 CH Utrecht, The Netherlands; 5 Institute of Meteorology and Climate Research, Karlsruhe Institute of Technology, 82467 Garmisch-Partenkirchen, Germany; 6 Centre for Ecology and Hydrology, Edinburgh, EH26 0QB UK; 7 Department of Forest Ecology and Management, Swedish University of Agricultural Sciences, SE901 83 Umeå, Sweden; 8 Natural Resources Institute Finland (Luke), FIN-00790 Helsinki, Finland; 9 Department of Earth Sciences, University of Gothenburg, SE405 30 Gothenburg, Sweden; 10 Institute of Earth Sciences, National Center for Scientific Research (CNRS) and University of Orléans, 45100 Orléans, France; 11 UNESCO Chair of Environmental Dynamics and Climate Change, Yugra State University, Khanty-Mansiysk, 628012 Russia; 12 Atmospheric Composition Research, Finnish Meteorological Institute, FIN-00101 Helsinki, Finland; 13 Department of Botany, Institute of Ecology and Earth Sciences, University of Tartu, 51014 Tartu, Estonia; 14 Everglades Wetland Research Park, Kapnick Center, Florida Gulf Coast University, Naples, FL 4940 USA; 15 Institute of Plant Ecology, Justus Liebig University Giessen, 35392 Giessen, Germany; 16 University College Dublin (UCD) School of Biology and Environmental Science, UCD Earth Institute, Dublin 4, Ireland; 17 Department of Plant Physiology, Institute of Agricultural and Environmental Sciences, Estonian University of Life Sciences, 51014 Tartu, Estonia; 18 Estonian Fund for Nature, 51014 Tartu, Estonia; 19 School of Geographical Sciences, University of Bristol, Bristol, BS8 1SS UK; 20 Department of Primary Industries, Parks, Water and Environment, Tasmanian Government, Hobart, TAS 7001 Australia; 21 Department of Agricultural Production, College of Agricultural and Environmental Sciences, Makerere University, 7062 Kampala, Uganda; 22 Hydrosystems and Bioprocesses Research Unit, National Research Institute of Science and Technology for Environment and Agriculture (IRSTEA), 92160 Antony, France; 23 Grupo de Investigación Aplicada al Medio Ambiente, Corporacion Universitaria Lasallista, Caldas, 51 118 Colombia; 24 Forest Resource Environment Development and Conservation Association, Yangon, 0951 Myanmar

Correction to: *Nature Communications* 10.1038/s41467-018-03540-1, published online: 19 March 2018.

The original version of this Article contained an error in the first sentence of the Acknowledgements section, which incorrectly referred to the Estonian Research Council grant identifier as “PUTJD618”. The correct version replaces the grant identifier with “PUTJD619”. This has been corrected in both the PDF and HTML versions of the Article.

