# Supplementary material for: Nitrogen-rich organic soils under warm well-drained conditions are global nitrous oxide emission hotspots
Source: Nat Commun. 2018 Mar 19;9:1135. doi: 10.1038/s41467-018-03540-1 (PMC5859301; doi:10.1038/s41467-018-03540-1)
Supplement: Supplementary file 1 — Supplementary Information [file 41467_2018_3540_MOESM1_ESM.pdf]

Supplementary Information for Pärn et al. „Nitrogen-rich organic soils under warm well-drained conditions are global nitrous oxide emission hotspots“

Supplementary Table 1. Average ± standard error N_2O emission from organic soils of the world, ug N_2O-N m^{-2} h^{-1}, according to drainage class and Köppen climate group. A, tropical; C, temperate; D, boreal.

	**A**	**C**	**D**
**Natural**	14.2±2.4	0.87±0.22	0.72±0.49
**Drained**	165±15	18.0±2.5	27.3±4.3

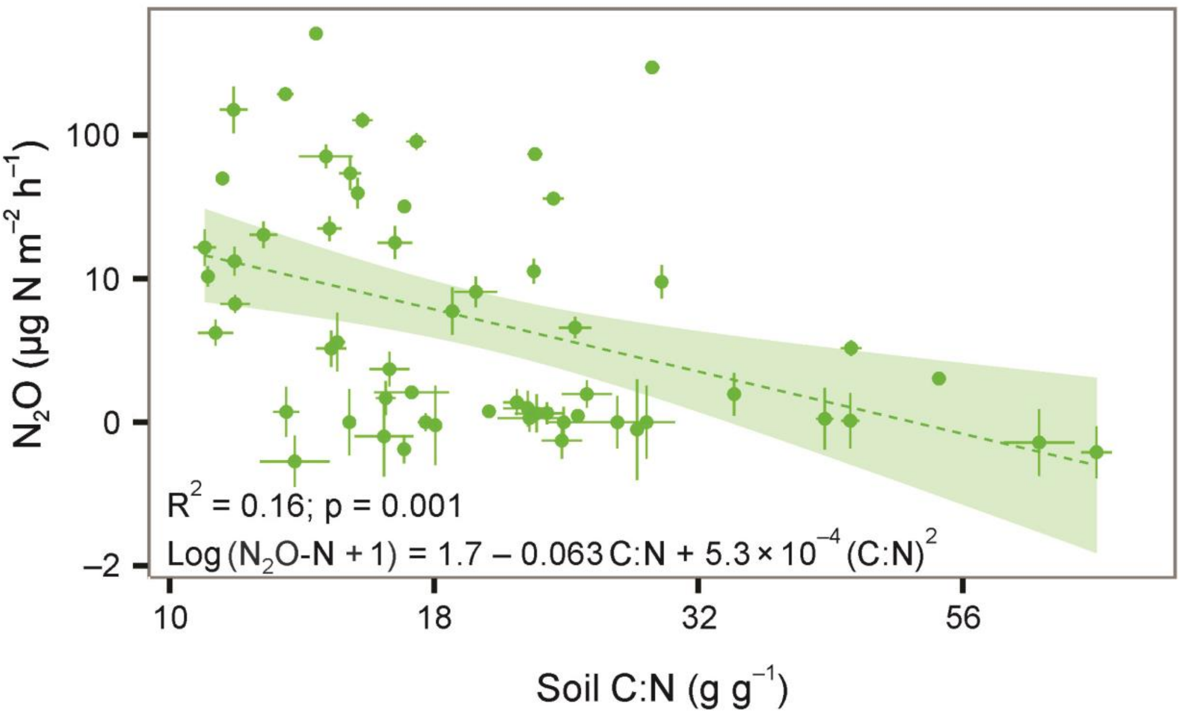

Supplementary Fig. 1. Relationship between site-mean N_2O fluxes +/– s.e.m. and C:N ratio. N=58.
