# Supplementary material for: Nitrogen-rich organic soils under warm well-drained conditions are global nitrous oxide emission hotspots
Source: Nat Commun. 2018 Mar 19;9:1135. doi: 10.1038/s41467-018-03540-1 (PMC5859301; doi:10.1038/s41467-018-03540-1)
Supplement: Supplementary file 3 — Description of Additional Supplementary Files [file 41467_2018_3540_MOESM3_ESM.pdf]

Description of Additional Supplementary Files

File Name: Supplementary Data 1
Description: Locations, dates, measurements of soil chemistry and physics, and N2O fluxes used in the analyses.
